# Pancreatic cancer driver mutations are targetable through distant alternative RNA splicing dependencies

**DOI:** 10.18632/oncotarget.27901

**Published:** 2021-03-16

**Authors:** Ryan R. Kawalerski, Steven D. Leach, Luisa F. Escobar-Hoyos

**Affiliations:** ^1^Medical Scientist Training Program, The Johns Hopkins University School of Medicine, Baltimore, MD 21205, USA; ^2^Departments of Molecular and Systems Biology, Surgery, and Medicine, Dartmouth Geisel School of Medicine and Norris Cotton Cancer Center, Lebanon, NH 03766, USA; ^3^Department of Therapeutic Radiology, Yale University, New Haven, CT 06513, USA; ^4^Department of Molecular Biophysics and Biochemistry, Yale University, New Haven, CT 06513, USA; ^5^Department of Pathology, Stony Brook University Renaissance School of Medicine, Stony Brook, NY 11794, USA

**Keywords:** pancreatic cancer, RNA splicing, targeted therapy, KRAS, TP53

## Abstract

Pancreatic ductal adenocarcinoma (PDAC), the most common histological subtype of pancreatic cancer, has one of the highest case fatality rates of all known solid malignancies. Over the past decade, several landmark studies have established mutations in *KRAS* and *TP53* as the predominant drivers of PDAC pathogenesis and therapeutic resistance, though treatment options for PDACs and other tumors with these mutations remain extremely limited. Hampered by late tumor discovery and diagnosis, clinicians are often faced with using aggressive and non-specific chemotherapies to treat advanced disease. Clinically meaningful responses to targeted therapy are often limited to the minority of patients with susceptible PDACs, and immunotherapies have routinely encountered roadblocks in effective activation of tumor-infiltrating immune cells. Alternative RNA splicing (ARS) has recently gained traction in the PDAC literature as a field from which we may better understand and treat complex mechanisms of PDAC initiation, progression, and therapeutic resistance. Here, we review PDAC pathogenesis as it relates to fundamental ARS biology, with an extension to implications for PDAC patient clinical management.

## INTRODUCTION

### PDAC epidemiology and treatment

Pancreatic ductal adenocarcinoma (PDAC) accounts for approximately 90% of all tumors of the pancreas, while the remaining 10% is comprised of predominantly pancreatic neuroendocrine tumors [1]. According to the most recent Surveillance, Epidemiology, and End Results (SEER) Program data, pancreatic cancer remains one of the deadliest solid malignancies in the United States, with a five-year survival of approximately 10% [2]. Routine screening is not practiced for early detection of pancreatic tumors, although high-risk patients with familial pancreatic cancer or known germline cancer-predisposing syndromes, accounting for 5–10% of all pancreatic cancer patients, may benefit from pancreatic screening and germline mutation testing [3–5].

Current therapy for PDAC patients includes surgical resection with adjuvant chemoradiation, increasing 5-year patient survival to approximately 20%, by some estimates [6, 7]. For over 80% of patients, however, PDAC is diagnosed as either borderline resectable, locally advanced, or metastatic disease, limiting eligibility for surgery [8–11]. Patients receiving systemic medical therapy either independent of surgery or in the adjuvant setting see significant yet minimal improvements in survival, though treatment options are often limited by patient tolerance [12–14]. While some PDACs harboring susceptibility-conferring mutations (e.g., *BRCA1/2*, *ATM*) are treatable via targeted medical approaches, such as poly (ADP-ribose) polymerase inhibition for PDACs with DNA repair gene mutations, these constitute a minority of all PDACs [15–17]. Even the most successful systemic medical treatments, gemcitabine plus nab-paclitaxel or FOLFIRINOX (a combination of 5-fluorouracil, leucovorin, irinotecan, and oxaliplatin), have demonstrated only a modest improvement in median patient survival of about 2-4 months beyond the gemcitabine control arm median survival of about 6 months [18–20]. Nevertheless, there is strong evidence suggesting a role for neoadjuvant systemic therapy to improve resectability of borderline resectable lesions [9, 21, 22].

### PDAC driver mutations and non-mutational driver-phenocopying mechanisms

Non-hereditary PDAC, accounting for about 90% of cases, is predominantly characterized by a well-established progression of mutational burden beginning with activating point mutations in the *KRAS* gene (about 90% of PDACs) [23–25]. Mutations in *KRAS* are often accompanied by secondary mutations, most commonly in tumor protein p53 (*TP53*, > 60% of cases), cyclin-dependent kinase inhibitor 2A (*CDKN2A*), and mothers against decapentaplegic homolog 4 (*SMAD4*) genes, conferring unique advantages to PDACs in therapy resistance and tumor aggression [26, 27]. Recent evidence via novel small molecule intervention and genetic ablation has shown that loss of oncogenic *KRAS* function, for example, is prone to initial tumor volume loss followed by tumor regrowth, either as a consequence of cancer cell heterogeneity in *KRAS* dependency or the presence of highly *KRAS*-dependent cells harboring the ability to undergo a stress-induced clonal escape mechanism mediated through advantageous functional alterations [28–32]. Epigenetic, metabolic, and immuno-modulatory processes have all been implicated in drug resistance and tumor maintenance in *KRAS*-mutant PDACs [33–37]. This suggests that even the most potent anti-KRAS targeted therapies are susceptible to mechanisms of therapy resistance.

Over the past decade, a wealth of information on PDAC RNA expression has contributed to a rapidly advancing understanding of the mechanisms by which these tumor cells may harbor treatable characteristics, either dependent or independent of tumor mutational status. Many studies of human tumor samples have led to a growing consensus on a two-subtype transcriptomic disease model described by the ‘Basal-like’ and ‘Classical’ gene signatures, which have been shown to correlate well with systemic therapy response, tumor aggression, and patient survival [38–46]. Work is ongoing to describe genetic characteristics of the PDAC stromal compartment, though early studies have shown a strong relationship between disease severity and stromal cell gene expression [39, 47, 48]. Even after subtyping PDACs by gene expression and mutational status, there still exists substantial variety in tumor therapy response and cellular characteristics in the preclinical setting [43, 44, 49], as recently reviewed in Du et al. [50]. Thus, there are likely other mechanisms, either epigenetic or otherwise hidden in summary gene expression data, by which tumors are initiated, maintained, and able to evade therapy that must be uncovered to effectively treat PDACs from several dependency-inspired angles.

### Alternative RNA splicing in normal and cancer cells

#### Alternative RNA splicing biology/functions

As mentioned above, recent literature has focused on using gene expression data to characterize PDACs, such as for determination of tumor transcriptomic subtypes, evaluation of potential disease biomarkers, and discovery of novel targetable disease mechanisms. Concurrently, substantial work has been conducted to describe the epigenetic, proteomic, and broadly metabolic characteristics of the disease. Nevertheless, there exists a comparable lack of investigation into alternative RNA splicing (ARS), an extremely plastic genetic control mechanism by which cells monitor and respond to stress, regulate gene expression, and influence intra- and inter-cell communication [51–57].

Alternative RNA splicing is a choreography by which RNA is processed to expand the protein diversity of eukaryotic organisms, via activity of the spliceosome, an RNA-protein complex. Exons, introns, and other noncoding RNA segments are recognized by RNA binding proteins (RBPs, including SR proteins and hnRNPs, among others) at conserved cis-regulatory RNA sequences to promote or suppress – in a summative fashion – RNA segment retention in the final mRNA product [58–60]. Estimates suggest that most genes with multiple exons undergo ARS to produce multiple distinct protein isoforms [61], and significant variation exists across tissue types as to the predominance of a given isoform, likely due to tissue-specific RBP expression and conditional RBP activity [62].

Several studies have posited that most alternative splicing events seen in next generation sequencing studies result from noisy aberrant splicing, leading to non-functional protein products [63–65]. Nevertheless, there is a growing basis of evidence for this noise and also for regulated diversification of isoform expression specifically contributing to organism and tissue development, normal cellular physiology, and pathology that might provide insight into cancer pathophysiology and therapy development [66, 67]. An active field of study is centered on the development of novel programmatic methods for assessing expression of annotated or novel protein isoforms using RNA sequencing platforms, though many investigators have also found success in home-brewed pipelines for analysis of this data [68–72]. This, along with the development of reliable long-read RNA sequencing technologies to enable precise quantification of transcripts at the whole mRNA level as well as continually advancing paired-end RNA sequencing at the single cell level, positions studies of ARS at a uniquely opportune time to uncover meaningful biochemical knowledge necessary for biomedical advancement.

### ARS mechanisms in cancer pathogenesis

ARS has been implicated in the initiation and maintenance of solid and non-solid malignancies [73–75]. There are several ways that ARS may be modified in cancer, including mutations in *cis*-regulatory RNA sequences, splicing protein post-translational modifications, and alterations in splicing protein primary sequences as well as expression levels, as reviewed in Escobar-Hoyos et al. [76]. While recent studies have demonstrated that RBPs known to interact with the spliceosome and globally alter ARS are mutated at a low rate in PDACs and two of the most common PDAC precursor lesions, pancreatic intraepithelial neoplasia and intraductal papillary mucinous neoplasm [77, 78], mechanistic understanding of their role in pathogenesis of PDACs and other solid tumors is extremely limited. Furthermore, there is a notable dearth of knowledge on the splicing-regulatory roles of the most commonly mutated genes in PDAC, *TP53* and *KRAS*.

To address this lack of knowledge, we recently uncovered a novel mechanism by which PDACs with *KRAS* and *TP53* mutations (a combination found in most PDACs) promote cancer pathogenesis via modified splicing of GTPase-activating protein (GAP) mRNAs and subsequent amplification of KRAS signaling [32]. Increased *hnRNPK* expression downstream from mutant p53 leads to increased retention of cytosine-rich exons in GAP mRNAs, thus leading to dysfunctional GAPs that are limited in their ability to transition KRAS from its active GTP-bound state to its inactive GDP-bound state. In the study, we show that *KRAS*- and *TP53*-mutant PDACs are selectively susceptible to spliceosome inhibition using H3B-8800 (an inhibitor of the SF3B complex, critical in spliceosome function and currently in phase I clinical trials) [79, 80], induction of *hnRNPK* functional loss, and correction of the cytosine-rich GAP splicing alterations using targeted oligonucleotide delivery. Thus, through this oncogenic dependency, it is likely that PDAC cells with both *KRAS* and *TP53* mutations could be targeted in the clinic, though more work must be conducted to evaluate whether or not these findings of efficacy will translate to studies on primary human pancreatic tumors. Importantly, *TP53* and *KRAS* mutations commonly co-occur in several solid malignancies, including lung adenocarcinoma and colorectal cancer, opening the possibility that tumors harboring this combination of mutations might also carry the same ARS oncogenic mechanisms and therapeutic susceptibilities as *KRAS*/*TP53*-mutant PDACs [81, 82].

Others have also demonstrated that aberrant mRNA splicing in PDACs may generate meaningful tumor biomarkers while also contributing to tumor progression and drug resistance [83–91]. For example, CD44, a cell-surface glycoprotein that undergoes extensive splicing of its 20 exons, is differentially spliced between PDAC and normal pancreas tissue [88]. Furthermore, expression of the ‘standard’ CD44 isoform (CD44s) as opposed to ‘variant’ forms (CD44v) is strongly associated with an epithelial-to-mesenchymal transition process in PDAC cells, and inclusion of variant exons v3 and v6 in the CD44 mRNA product is uniquely associated with cancer metastasis [89, 90]. Other studies have shown that ARS alterations in PDACs strongly target extracellular matrix components [86, 91]. In another instance, the pyruvate kinase (*PKM*) gene was shown to predominantly produce the PKM2 isoform in gemcitabine-resistant PDAC cells, for which metabolic gemcitabine sensitivity could be restored following targeted antisense oligonucleotide delivery to promote production of the alternative PKM1 isoform [85]. Some studies have recently presented large-scale analyses of PDAC splice variant expression, though there is yet substantial work needed to harmonize these findings with the growing biomedical understanding of PDAC as well as routine clinical practice [84, 86].

### Treating alternative RNA splicing defects

#### ARS dependencies are targetable in PDACs, other malignancies, and non-malignant diseases

Both targeted oligonucleotide delivery and small molecule inhibition of the spliceosome have been shown to be effective at treating several carcinomas in preclinical models, in addition to other splicing-focused therapies [32, 73]. Targeted oligonucleotides can be quickly generated to correct mutated, improperly spliced, or otherwise defective mRNA products with high specificity and efficacy when properly delivered to target cells [92]. Perhaps the most famous example of dysfunctional mRNA gene product correction is that of nusinersen (Spinraza), approved by the FDA in 2016 as the first medical treatment for spinal muscular atrophy, offering strong proof-of-concept for clinical efficacy of this treatment modality [93, 94]. New oligonucleotide delivery methods have improved clinicians’ abilities to deliver therapeutic doses into difficult-to-reach tumors, like PDACs, taking advantage of tumor microenvironmental factors such as acidic pH, for example [75, 95, 96]. As an alternative method for targeting ARS pathogenic mechanisms, small molecule spliceosome modulators have demonstrated efficacy in treating cancers preferentially susceptible to spliceosome dysfunction [80], such as those harboring heterozygous mutations in SF3B1, a critical component of the spliceosome for which complete functional loss is synthetic lethal [97].

The therapeutic utility of a targeted anti-cancer drug relies heavily on rapid and accurate tumor profiling, often in practice requiring immunohistochemical staining of fixed tissue to inform clinical decision-making. Currently, gene expression and subtyping methods for PDACs, though holding high potential for clinical translation, rely on a days-to-weeks-long approach involving RNA quantification and subsequent analysis. Evaluation of splicing changes, however, may be feasibly conducted through simple RT-PCR methods, enabling highly specific and rapid identification of actionable ARS dependencies. Our recent work, together with the corpus of evidence supporting clinical translation of ARS events for cancer therapy, provides a compelling vision of future oncology practice involving targeted ARS tumor profiling through scalable RNA amplification and visualization methods [98].

## FUTURE PROSPECTS

Advancements in high-throughput RNA sequencing technologies over the past decade have led to substantial growth in the understanding of RNA splicing in cancer, and specifically PDAC. While ours and other studies have established strong connections between well-studied molecular alterations and splicing changes, several fundamental questions remain unanswered about the role of ARS in PDAC and, more broadly, cancer pathogenesis as whole. Evidence is limited on the capacity of ARS alterations to phenocopy mutational signatures as well as the role of ARS in cellular transformation downstream from bona fide cancer-initiating genomic mutations. The recent expanse of data on non-malignant pancreatic tumor co-conspirator cells – including cancer-associated fibroblasts and tumor-infiltrating immune cells, for example – further opens an exciting opportunity to understand how ARS might contribute to tumor cell immune evasion as well as the drastic desmoplastic collagen deposition in PDACs. Further investigation of these mechanisms may likely translate to clinically effective therapeutics, in addition to enabling a well-rounded understanding of cancer pathogenesis, maintenance, drug resistance, and immune evasion.
